# Innovative use of gram-positive enhancer matrix particles and affinity peptides in a vaccine against Coxsackievirus B3

**DOI:** 10.1080/21505594.2025.2481657

**Published:** 2025-04-02

**Authors:** Shaoju Qian, Ruixue Li, Guanyu Chen, Yinghua Ma, Xuehan Zhang, Zhou Tang, Yihang Song, Zhishan Xu, Zihan Zhang, Yeqing He, Xingyi Zhang, Shuao Lu, Zishan Yang, Xiangfeng Song, Wenfa Yu, Lili Yu

**Affiliations:** a School of Basic Medical Sciences, Xinxiang Medical University, Xinxiang, 453003, China; b Department of Otolaryngology, The First Affiliated Hospital of Xinxiang Medical University, Xinxiang, 453003, China; c Xinxiang Key Laboratory of Tumor Vaccine and Immunotherapy, School of Basic Medical Sciences, Xinxiang Medical University, Xinxiang,453003, China; d Xinxiang Engineering Technology Research Center of Immune Checkpoint Drug for Liver-Intestinal Tumors, Henan 453003, China

**Keywords:** Coxsackievirus B3, vaccine, gram-positive enhancer matrix, affinity peptides

## Abstract

Viral myocarditis (VM) is an inflammatory disease posing a serious threat to public health, with various viral pathogens contributing to its pathogenesis. Coxsackievirus B3 (CVB3) is the most frequently implicated causative agent and has been extensively studied because of its high prevalence and severity. No specific therapeutic interventions for VM exist, and vaccine development has encountered substantial challenges. Therefore, we aimed to develop a novel CVB3 mucosal vaccine as a preventive strategy against VM. Gram-positive enhancer matrice (GEM) particles serve as innovative mucosal vaccine adjuvants and antigen delivery systems that enhance antigen immunogenicity by facilitating effective mucosal immune responses. In this study, GEM particle display technology was used to develop two novel CVB3 vaccines: (1) a GEM particle-based vaccine displaying the CVB3 capsid protein VP1 via a PA anchor protein (GEM-PA-VP1), and (2) a GEM particle-based vaccine displaying VP1 via the FcSP peptide (GEM-Fc-VP1). Both GEM-PA-VP1 and GEM-Fc-VP1 vaacines significantly elevated levels of specific IgG, IgG1, IgG2a, sIgA and neutralizing antibodies in a mouse model, along with enhanced secretion of Th1- and Th2-associated cytokines, compared to controls. Notably, GEM-Fc-VP1 demonstrated superior immunogenicity compared with that of GEM-PA-VP1, evidenced by higher antibody titres and cytokine responses. In challenge protection experiments, both vaccines significantly improved survival rates, reduced myocardial enzyme levels, and decreased inflammatory cell infiltration in myocardial tissue, with GEM-Fc-VP1 exhibiting greater efficacy. These findings establish a foundation for the development of a safe and effective CVB3 candidate vaccine and provide novel insights into the potential of peptide-mediated subunit vaccine approaches.

## Introduction

Coxsackievirus B3 (CVB3), a member of the genus *Enterovirus* within the Picornaviridae family, is a highly contagious pathogen with a global prevalence [1,2]. CVB3 is the principal causative agent of viral myocarditis (VM), that is a major cause of heart failure in children and adolescents, posing a serious threat to public health [3,4]. The mortality rate of VM in young adults can reach up to 21%, with acute VM often progressing rapidly and resulting in high mortality [5]. By 2017, approximately 1.8 million new cases of VM were reported annually [5]. The current treatment methods primarily provide supportive care [4,6], with no specific therapies available to target CVB3 infection, highlighting the urgent need for effective preventive measures.

The CVB3 capsid protein VP1, the primary protective antigen, contains multiple T- and B-cell epitopes and exhibits strong immunogenicity, making it an ideal candidate for vaccine development [7,8]. The neonatal Fc receptor (FcRn), which transports immunoglobulin G (IgG), is widely expressed in epithelial cells and macrophages and plays a critical role in immune processes by mediating IgG transport and maintaining IgG levels in the bloodstream [9]. Previous studies showed a significant association between the FcRn and intestinal coronavirus infection, where Fc fusion proteins traverse mucosal epithelial cells via FcRn-mediated transcytosis, eliciting both local mucosal and systemic immune responses [10,11]. Since CVB3 infects via mucosal pathways, inducing a mucosal immune response could represent a crucial advancement in the prevention and treatment of CVB3 infections.

In this study, we explored a novel mucosal vaccine delivery system using gram-positive enhancer matrix (GEM) particles. GEM particles, approximately 1 µm in diameter, can traverse the nasal mucosa, effectively triggering mucosal immune responses [12]. The GEM system incorporates exogenous antigen proteins into the peptidoglycan particles of *Lactococcus lactis* through fusion them with a protein anchor (PA), thereby forming antigen-presenting particles [13,14].

The GEM-PA system has been previously applied in various animal models [15–17], eliciting robust immune responses upon mucosal administration and providing protective effects against parasites [18], bacteria [19], and viruses [20]. Despite their potential, the application of PA-fusion proteins is limited due to challenges such as low solubility, suboptimal expression levels, and potential toxicity.

To overcome these challenges, we introduced affinity peptide technology as a substitute for the PA system. We used the affinity peptide FcSP, which specifically binds to the Fc fragment of IgG [21], in combination with the VP1-Fc fusion protein. Fc fusion protein offers several advantages, including stable protein folding, an extended half-life, enhanced immune functionality, efficient expression, ease of purification, and reduced burden on host cells [22,23]. In this study, FcSP was used to bind the VP1-Fc fusion protein to GEM particles, effectively preserving the structure and function of the antigen, thereby eliciting a robust immune response.

The study aimed to evaluate the efficacy of GEM particles as a delivery vector for CVB3 mucosal vaccines and assess its potential for preventing CVB3 infection. We analysed the characterization and effects of the GEM-PA-VP1 and GEM-Fc-VP1 on infection symptoms, inflammatory responses, and histopathological changes in a mouse model of VM. Furthermore, we assessed the effects of the vaccine on neutralizing antibody titres and survival rates. This study provides novel insights and strategies for the development of effective preventive measures and therapeutic interventions for VM.

## Materials and methods

### Strains, plasmid, and antibodies

*Lactococcus lactis* MG1363 and *Escherichia coli* DH10Bac strains were sourced from Zoman Biotechnology (Beijing, China), whereas the pcDNA3.1(+) plasmid was maintained as a stock in our laboratory. *E. coli* DH10Bac was cultured in Luria – Bertani (LB) medium under standard growth conditions. For immunodetection, an anti-His monoclonal antibody (dilution 1:2000) was employed, along with horseradish peroxidase (HRP)-conjugated goat anti-mouse secondary antibody (dilution 1:500). Additionally, anti-mouse IgG, IgG1, IgG2a and sIgA antibodies (each diluted 1:2000) were sourced from ABclonal (Wuhan, China).

### Recombinant plasmid construction

The construction of the recombinant plasmids was based on the coding sequences of CVB3 *VP1* (GenBank accession no. GQ329748.1), protective antigen (PA; GenBank accession no. U17696.1), and mouse IgG Fc (GenBank accession no. V00798.1), retrieved from the NCBI nucleotide database. The *VP1* coding sequence was optimized to include an albumin (ALB) signal peptide and subsequently fused with either the PA or mouse IgG Fc gene to generate the fusion constructs VP1-PA and VP1-Fc, respectively. These fusion constructs were cloned into the pUC57 vector, producing the recombinant vectors pUC57-VP1-PA and pUC57-VP1-Fc, with the synthesis performed by GenScript (Nanjing, China).

Using these recombinant vectors as templates, the VP1, PA, and Fc coding sequences were amplified through polymerase chain reaction (PCR) with specific primers (Table 1). The fusion genes VP1-PA and VP1-Fc were then digested using the restriction enzymes *Kpn*I and *Xho*I, and the resulting fragments were ligated into the pcDNA3.1(+) expression vector using T4 DNA ligase, generating the expression plasmids pcDNA3.1-VP1-PA and pcDNA3.1-VP1-Fc.

**Table 1.** Primers used in this study.

Primer name	Sequence (5′- 3′)
VP1-F	AATGGTACCATGAAGTGGGTAACCTTTATTTTCCC
VP1-R	AAACGCCCCAGTATTGGTCAGGCGGGGGTGGGTCCGGAG
PA-F	TGACCAATACTGGGGCGTTTGGCGGGGGTGGGTCCGGA
PA-R	AATCTCGAGTTTGATTCTCAGGTACTGCCCTATT
Fc-F	TGACCAATACTGGGGCGTTTGGCGGGGGTGGGTCCGGA
Fc-R	AATCTCGAGTTTACCCGGAGTCCGGGA

### Protein expression

Chinese hamster ovary (CHO) cells (Servicebio, China, STCC40001-1P) were cultured in DMEM/F-12 medium supplemented with 10% foetal bovine serum (FBS) (Biological Industries, Israel, 04–121-1A). For transfection, recombinant plasmids (pcDNA3.1-VP1-PA or pcDNA3.1-VP1-Fc) were complexed with Lipofectamine 3000 in Opti-MEM, a serum-free medium, to form DNA-liposome complexes. These complexes were introduced into CHO cell cultures and incubated at 37 °C for 4–6 h. Following incubation, the cells were thoroughly washed with phosphate-buffered saline (PBS) to remove residual complexes. His-tagged proteins were then purified using nickel nitrilotriacetic acid (Ni – NTA) affinity chromatography. Protein expression and purification efficiency for VP1-PA and VP1-Fc were evaluated through 10% sodium dodecyl sulphate – polyacrylamide gel electrophoresis under denaturing conditions. After electrophoresis, proteins from the supernatants and cell debris were transferred onto nitrocellulose membranes (Millipore, USA, Cat. No. ISEQ00010). Detection was performed using an anti-His monoclonal antibody (1:2000) as the primary antibody, followed by a HRP-conjugated goat anti-mouse secondary antibody (1:500). Western blotting was performed to confirm protein expression, and indirect immunofluorescence assays (IFA) were used to validate successful transfection and expression in the CHO cells.

### Preparation of GEM particle vaccine displaying VP1 protein

Freshly cultured *Lactococcus lactis* MG1363 cells were harvested from M17 medium after an overnight incubation at 30°C and then washed with sterile PBS. Cell lysis was induced by boiling the bacteria in 10% trichloroacetic acid for 30 min. Following lysis, the cells were washed thrice with PBS to remove residual trichloroacetic acid, and bacterial enumeration was performed, defining 2.5 × 10^9^ particles as one unit (U). The resulting particles were subjected to vacuum drying, followed by gold coating for analysis via transmission electron microscopy (TEM) to assess their morphology. The generated GEM particles were stored at − 80 °C for future use.

**Figure 1.**
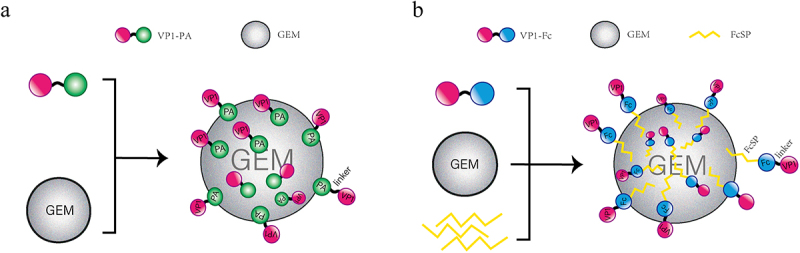
Design of the two vaccine structures based on GEM particles: GEM-PA-VP1 (a) and GEM-Fc-VP1 (b). (a) The VP1-PA fusion protein is displayed on the surface of GEM particles. In the GEM-PA-VP1 vaccine, the PA anchor is utilized to present CVB3 VP1 on the surface of GEM particles. (b) The VP1-Fc fusion protein is displayed on the surface of GEM particles via the mediation of the FcSP polypeptide. In the GEM-Fc-VP1 vaccine, the PA anchor is replaced by the FcSP affinity peptide, which binds the VP1-Fc fusion protein to GEM particles.

To prepare GEM-PA-VP1 antigen display particles (Figure 1a), GEM particles (2.5 × 10^9^ particles/mL) were incubated with the VP1-PA fusion protein and gently stirred at 25°C for 2 h, and then centrifuged at 2,000 rpm for 15 min at 4 °C. The resulting pellet was washed with PBS.

To prepare GEM-Fc-VP1 antigen display particles (Figure 1b), GEM particles were treated with N-hydroxysuccinimide (NHS) and 1-ethyl-3-(3-dimethylaminopropyl) carbodiimide (EDC) solutions (Sigma, USA) to activate carboxyl groups for peptide conjugation. These activated particles were then incubated with the affinity peptide HWRGWV-FcSP (Synpeptide, China), diluted in PBS, and subjected to centrifugation at 12,000 rpm for 2 min. After being washed twice with PBS, the particles were reconstituted in PBS and mixed with the VP1-Fc fusion protein. Following another centrifugation at 12,000 rpm for 5 min and subsequent washing with PBS-Tween (PBST) and PBS, the particles were resuspended in PBS.

GEM-PA-VP1 and GEM-Fc-VP1 particles were characterized by transmission electron microscopy, and their sizes and zeta potentials were measured by dynamic light scattering (DLS) and electrophoretic light scattering (ELS) methods as previously described [12,24]. The fusion protein, either PA-VP1 or Fc-VP1, was coupled with GEM particles and subsequently released from them, and it was quantified by the BCA protein assay as previously described [25]. The toxicity and proinflammatory properties of GEM-PA-VP1 and GEM-Fc-VP1 particles on RAW264.7 cells were evaluated as previously described [12].

### Immunization and sample collection

All animal procedures were conducted in accordance with the ethical guidelines approved by the Animal Ethics Committee of Xinxiang Medical University (approval no. XYLL-20240127) and adhered to the ARRIVE 2.0 guidelines andinternational standards for animal care and use.

For immunization, GEM-PA-VP1 or GEM-Fc-VP1 particles were mixed in equal proportions with Montanide™ IMS1313 N VG (Seppic, Paris, France). In total, specific-pathogen-free (SPF) male C57BL/6 mice (6–8-weeks-old, 20–22 g) were obtained from Henan Sbeck Biotechnology (Henan, China) and randomly divided into five groups . The groups received intranasal inoculations of PBS, GEM particles, VP1, GEM-PA-VP1, or GEM-Fc-VP1.

The immunization schedule comprised three phases: initial immunizations on days 1 to 3, booster immunizations from days 11 to 13, and final immunizations from days 21 to 23, with each phase spanning 3 consecutive days. Blood samples were collected on days 14, 28, and 42, and sera were separated, heat-inactivated at 56 °C for 30 min, and stored at −20 °C for subsequent analysis. To evaluate the immune response, body weights of CVB3-infected mice were recorded after the final immunization. On day 42 post-immunization, the mice were euthanized by carbon dioxide inhalation followed by cervical dislocation, and the spleen and heart tissues were collected for analysis. This euthanasia method is recognized for its rapidity and minimal distress to the animals.

### Detection of serum antibodies and cytokines

The concentrations of CVB3 VP1-specific antibodies, including IgG, IgG1, IgG2a, sIgA and neutralizing antibodies, were quantified using enzyme-linked immunosorbent assay (ELISA). For this, 96-well microplates (Corning-Costar, Corning, NY, USA) were coated overnight at 4 °C with 100 µL/well of purified CVB3 VP1 protein (50 µg/mL), expressed in *E. coli*, and dissolved in carbonate buffer. The plates were blocked with 5% skim milk washed thrice with PBS containing 0.05% Tween-20 (PBST). Then added the sample on the plate for incubation 30 min. Subsequently, the plates were incubated at 37 °C for 1 h with HRP-conjugated goat anti-mouse IgG, IgG1, IgG2a or sIgA antibodies, each diluted to 1:2000. Following incubation, the plates were washed again with PBST, and 100 µL of tetramethylbenzidine was added as a substrate. The reaction was stopped with 50 µL of 2N sulphuric acid (H₂SO₄), and the optical density was measured at 450 nm using an ELISA plate reader.

In parallel, the levels of interferon-gamma (IFN-γ), IL-4, tumour necrosis factor-alpha (TNF-α), IL-2, and IL-10 were measured using the indication mouse cytokine ELISA kit, respectively. To assess neutralizing antibody titres, a plaque reduction neutralization test (PRNT50) was performed. Serum samples were serially diluted and incubated with an equal volume of CVB3 virus at 37 °C for 1 h. The serum-virus mixtures (200 μL) were then added to the Vero cell monolayers and incubated at 37 °C for 1 h. After replacing the medium with 2% agarose, the cells were incubated for 4 days at 37 °C. Post-incubation, the cells were fixed with paraformaldehyde (MCE, USA) and stained with crystal violet (MCE, USA). Plaques were counted to determine the 50% neutralization titre (PRNT50), representing the serum’s neutralizing capacity.

### Assessment of myocardial enzyme activities and histopathological Hematoxylin & Eosin (HE) staining

After completion of the immunization protocol, the mice were anesthetized and challenged with CVB3 at a concentration of 1 × 10^4^ TCID_50_/mL. Each mouse was intranasally administered 100 µL of the viral suspension. At 7 days post-infection, the animals were euthanized, and myocardial tissue samples were collected for biochemical and histopathological analysis. To assess myocardial injury, the levels of creatine kinase (CK) and creatine kinase-MB (CK-MB), key markers of cardiac damage, were quantified using commercial enzyme assays (Solarbio, Shanghai). Myocardial tissues were analysed for TNF-α, IL-1β, and IL-6 to assess the inflammatory response. HE staining was used to assess infection-related damage, including mucosal injury, tissue degeneration, and inflammatory cell infiltration.

### Statistical analysis

All data are expressed as means ± standard deviation (SD) and were analysed using GraphPad Prism 8.0 (GraphPad Software, San Diego, CA). Statistical significance was determined by one-way analysis of variance (ANOVA), followed by Duncan’s multiple range test for post-hoc comparisons. A significance level of *p* < 0.05 was applied to identify statistically significant differences and highly significant differences were indicated by *p* < 0.01.

## Results

### Expression and identification of recombinant protein

**Figure 2.**
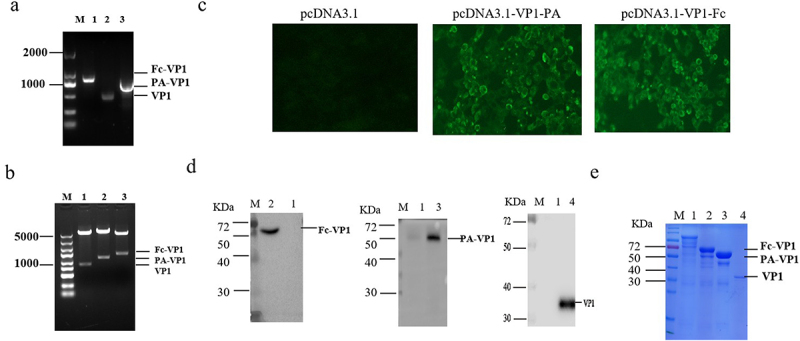
Construction of recombinant plasmids and their subsequent transfection and expression validation in cells. (a) PCR specific amplification the fragment of VP1, PA-VP1 and fc-VP1. (b) Identification of the recombinant plasmids by double enzyme digestion. M: Marker; Lane 1: pcDNA3.1-VP1; Lane 2: pcDNA3.1-PA-VP1; Lane 3: pcDNA3.1-Fc-VP1. (c) Expression of fusion proteins in CHO cells confirmed using immunofluorescence analysis. (d) Western blotting analysis of the fusion proteins. (e) SDS-page analysis of the purified fusion proteins. M: Marker;Lane 1: negative Control; Lane 2: Fc-VP1; Lane 3: PA-VP1; Lane 4: VP1.

To confirm the construction of recombinant plasmids, PCR amplification and restriction enzyme digestion were performed. PCR amplified the VP1-PA and VP1-Fc genes, producing expected DNA fragments, respectively (Figure 2a). The recombinant plasmids pcDNA3.1-PA-VP1 and pcDNA3.1-Fc-VP1 with the restriction enzymes KpnI and XhoI, which yielded a large fragment of the same size as pcDNA3.1 and a small fragment of the same size as the PA-VP1 (1521 bp) or Fc-VP1 (1635bp) genes, respectively (Figure 2b). The immunofluorescence assay (IFA) results for the PA-VP1 and Fc-VP1 proteins were positive, exhibiting bright green fluorescence, whereas the control cells lacked green fluorescence (Figure 2c).

The VP1-PA and VP1-Fc recombinant proteins were effectively expressed in CHO cells, as evidenced by Western blotting, and predominantly accumulated in the supernatant of the cell lysate, displaying molecular weights of approximately 55 and 59 kDa, respectively (Figure 2d).

Purification was performed using immobilized metal affinity chromatography (IMAC) on a Ni – NTA column, followed by desalting and concentration. The purified proteins were subsequently detected by SDS-PAGE (Figure 2e), confirming their successful expression and purification.

### Surface display of VP1 on GEM particles

**Figure 3.**
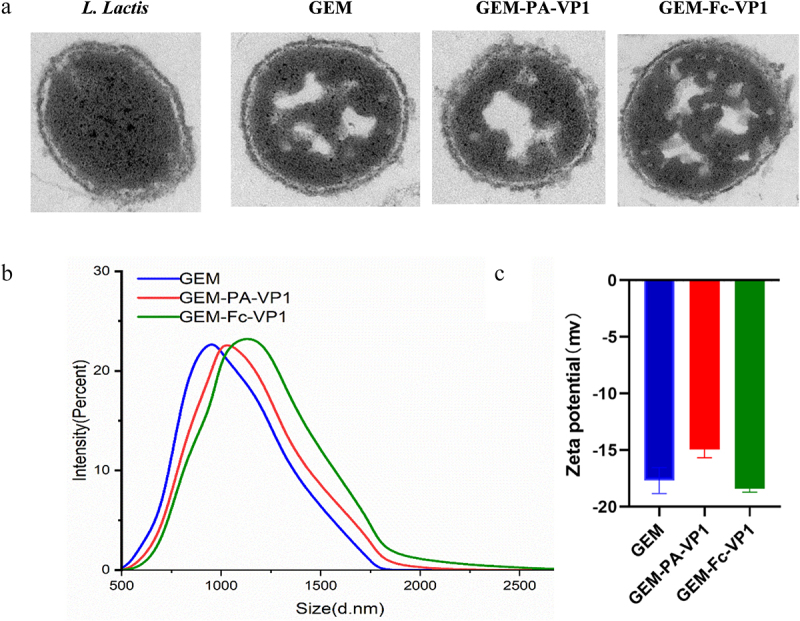
Display of recombinant proteins PA-VP1 and Fc-VP1 on GEM particles. (a) Transmission electron microscopy images of GEM, GEM-PA-VP1, and GEM-Fc-VP1 particle vaccines. (b, c) the size and zeta potential of GEM-PA-VP1 and GEM-Fc-VP1 particles detected by DLS and ELS methods using a zetasizer.

Using NHS and EDC as coupling agents, FcSP peptides were covalently bound to the surface of GEM particles, resulting in the formation of GEM-FcSP particles. The subsequent addition of VP1-Fc protein led to the formation of GEM-Fc-VP1 particle, with effective surface presentation of the VP1-Fc protein. Similarly, incubation of the VP1-PA protein with GEM particles led to the creation of GEM-PA-VP1 particle. TEM analysis revealed that GEM-PA-VP1 and GEM-Fc-VP1 exhibited a ring of flocculent material on their surface, whereas the surface of the GEM particle control appeared relatively smooth (Figure 3a). The average particle sizes of GEM nanoparticles measured using the Zeta sizer electronic particle size analyser were 955 nm. Compared to GEM, the average particle sizes of GEM-PA-VP1 and GEM-Fc-VP1 both increased to 1006 nm and 1181 nm. The Zeta potential shifted from −17.7 mV for GEM to −15.0 mV for GEM-PA-VP1 and −18.4 mV for GEM-Fc-VP1 (Figure 3b,c). These results indicate the efficient presentation of both VP1-PA and VP1-Fc proteins on the GEM particle surfaces.

### Characterization of GEM-PA-VP1 and GEM-Fc-VP1 particles

**Figure 4.**
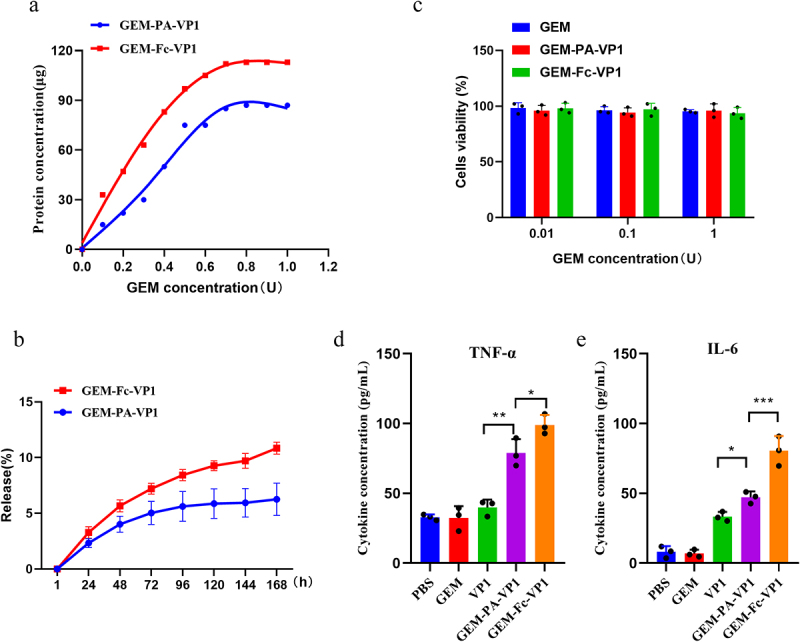
Characterization of GEM-PA-VP1 and GEM-Fc-VP1 particles. (a) Measurement of antigen loading efficiency on GEM particles by BCA protein assay. (b) Antigen release from GEM-PA-VP1 and GEM-Fc-VP1 was assessed by incubating in PBS (pH 7.4) for 0–168 h and protein content was quantified using a BCA protein assay. (c) Toxicity of GEM-PA-VP1 and GEM-Fc-VP1 particles to RAW264.7. (d, e) Expression of TNF-α and IL-6 in RAW264.7 after stimulated for 24 h. Data, derived from three parallel repeats per sample, are presented as mean ± SD (*p < 0.05, ** *p* < 0.01, ***p < 0.001).

To determine the amount of fusion protein bound on GEM particles, BCA protein analysis was performed. The analysis show that 1 U of GEM particles bind approximately 87 µg of PA-VP1 fusion protein and 113 µg of Fc-VP1 fusion protein (Figure 4a). Since fusion proteins are released during the first hour occurs during reconstitution, dilution, and application of the GEM, it is considered negligible for the vaccination studies. Consequently, the release curves were adjusted by subtracting the antigen released in the first hour. This adjustment resulted in a corrected total release of 6.25% for GEM-PA-VP1 and 10.84% for GEM-Fc-VP1 over 168 hours (Figure 4b). To assess potential toxicity of the GEM formulations, we incubated RAW264.7 cells with various GEM concentrations and evaluated cell viability. In a concentration range from 0.01 to 1 U, GEM-PA-VP1 and GEM-Fc-VP1 particles showed no detectable toxicity (Figure 4c). ELISA assay of cytokines showed that GEM-PA-VP1 particles and GEM-Fc-VP1 particles could significantly stimulate the expression of TNF-α and IL-6 in RAW264.7, and GEM-Fc-VP1 group was significantly higher than that of other groups (Figure 4d,e). These cytokines play a pivotal role in initiating and enhancing the activation of antigen-presenting cells, underscoring their immunological significance.

### Measurement of IgG, sIgA and neutralizing antibodies in immunized mice

**Figure 5.**
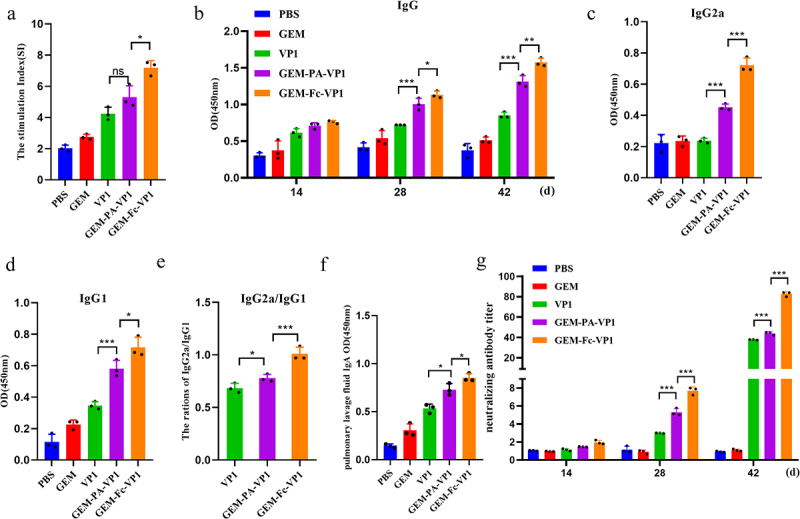
Effects of the GEM-PA-VP1 and GEM-Fc-VP1 vaccines on IgG levels, subtypes, neutralizing antibodies and SIgA in immunized mice. ELISA was used to measure the levels of various indices in mouse serum after the initial, booster, and final immunizations, respectively. (a) Spleen index of immunized mice. (b) Dynamics of IgG in serum samples from of immunized mice at 14, 28, and 42 days post immunization. (c, d) Detection of IgG2a, and IgG1 in serum samples of immunized mice at 42 days post immunization. (e) The ratio of IgG2a to IgG1. (f) sIgA levels in bronchoalveolar lavage fluid of immunized mice at 42 days post immunization. (g) Dynamics of neutralizing antibodies in serum samples of immunized mice at 14, 28, and 42 days post immunization. Data are presented as the mean ± SD.(*n* = 3 mice/group/time point, *p < 0.05, ** *p* < 0.01, ***p < 0.001).

To systematically assess immune responses elicited by bacterium-like particle vaccines, we quantitatively measured serum IgG antibodies and their subtypes (IgG1 and IgG2a) using ELISA. Serum samples were collected on days 14, 28, and 42 after the initial, booster, and final immunizations, respectively. The immunized groups demonstrated significant increases in the spleen index and total IgG levels compared with those in the control group (Figure 5a,b). Specifically, the GEM-Fc-VP1 group exhibited significantly higher levels of IgG1 and IgG2a, with an elevated IgG2a/IgG1 ratio, compared with those of the GEM-PA-VP1 group (Figure 5c-e). Moreover, the sIgA levels in bronchoalveolar lavage fluid were detected and found the GEM-Fc-VP1 group exhibited higher levels than the GEM-PA-VP1 group (Figure 5f). In the neutralizing antibody assay, both the GEM-PA-VP1 and GEM-Fc-VP1 groups produced significantly higher neutralizing antibody titres than those of the control group. The GEM-Fc-VP1 group achieved a titre of 1:84, significantly surpassing that of the GEM-PA-VP1 group (1:45) (Figure 5g). Collectively, these findings suggest that GEM-Fc-VP1 is more effective than GEM-PA-VP1 in promoting humoral immune responses, presenting a promising strategy for the prevention of VM.

### Detection of cytokine secretion by splenocytes in immunized mice

**Figure 6.**
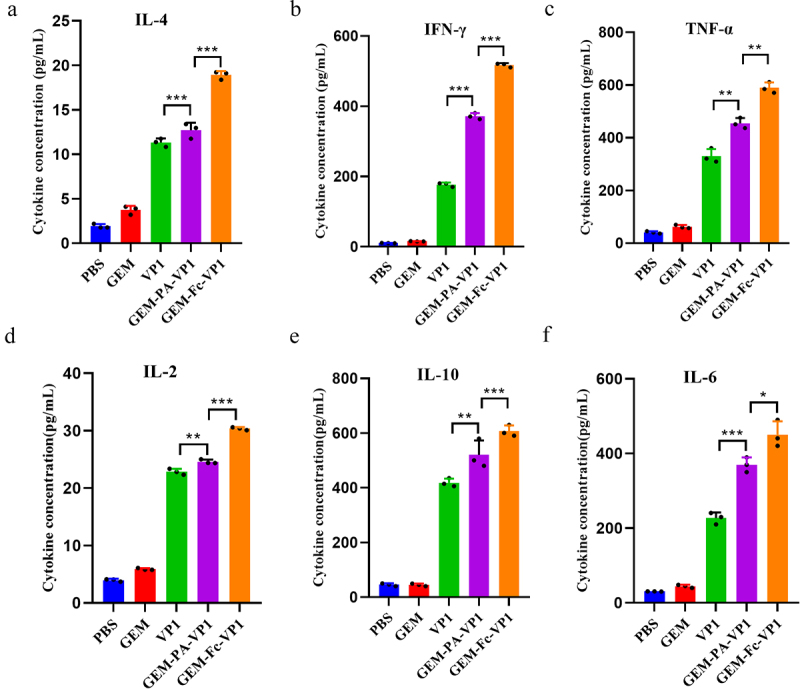
Detection of GEM-PA-VP1 and GEM-Fc-VP1 vaccines on cytokine secretion by mouse splenocytes. Splenocytes were harvested from the mice 42 days after immunization and stimulated with VP-1-specific antigen *in vitro*. The levels of IL-4 (a), IFN-γ (b), TNF-α (c), IL-2 (d), IL-6 (f), and IL-10 (e) were measured using ELISA. Data are presented as the mean ± SD. (*n* = 3 mice/group/time point, *p < 0.05, ** *p* < 0.01, ***p < 0.001).

As shown in Figure 6, the concentrations of IFN-γ, TNF-α, and IL-2 in splenocytes from mice immunized with GEM-PA-VP1 and GEM-Fc-VP1 were significantly elevated compared with those in the control group, signifying the induction of a robust T-helper (Th1) immune response. Notably, the GEM-Fc-VP1 group exhibited substantially higher concentrations of IFN-γ, IL-2 and TNF-α than those of the GEM-PA-VP1 group, underscoring its enhanced Th1 immunogenicity (Figure 6b-d). Furthermore, the GEM-Fc-VP1 group demonstrated significantly elevated levels of IL-4, IL-6 and IL-10, relative to both the control and GEM-PA-VP1 groups, reflecting concurrent activation of the Th2 pathway (Figure 6a,e,f). These data suggest that GEM-Fc-VP1 not only elicits a potent Th1 response but also stimulates a comprehensive Th2 immune profile, thereby offering superior immunological breadth and efficacy.

### Vaccine-induced immunity against CVB3 in challenged mice

**Figure 7.**
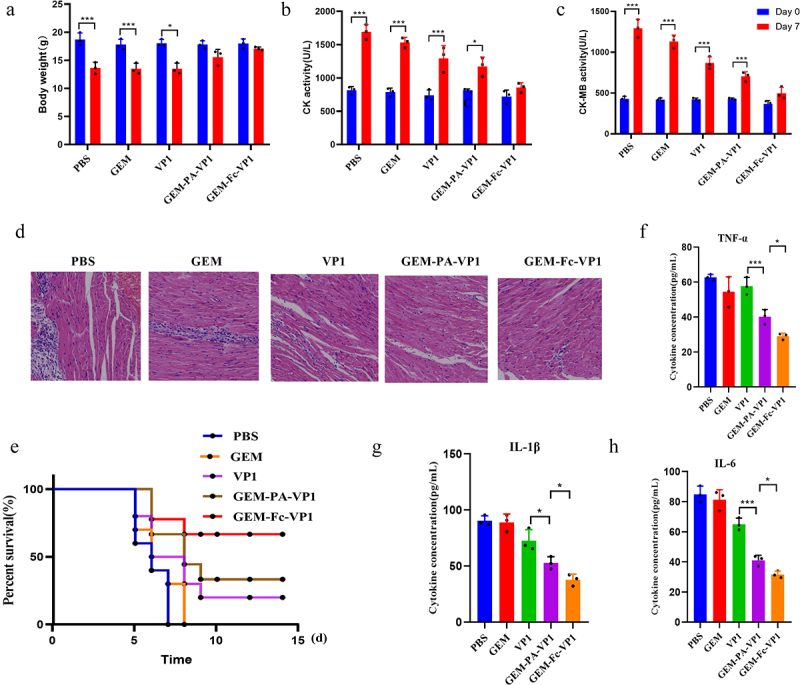
Enhanced immunoprotection against CVB3 infection by GEM-PA-VP1 and GEM-Fc-VP1 immunization. Mice were challenged with CVB3 (10^4^ TCID_50_) after the end of immunization. (a) The changes in average body weight of each group. (b, c) Detection of the serum myocarditis inflammation indexes CK and CK-MB enzyme in each group. (d) Hematoxylin and eosin stained myocardial tissue sections of mice at 7 days post-CVB3 challenge. (e) The survival rate of mice within 14 days post-CVB3 infection. (F-H) detection of TNF-α, IL-1β and IL-6 in myocardial tissues at 7 days post-CVB3 infection. Data are presented as the mean ± SD. (*n* = 3 mice/group/time point, *p < 0.05, **p < 0.01, ***p < 0.001).

To evaluate the immunoprotective efficacy of the particulate vaccine, mice were challenged with CVB3 (10^4^ TCID_50_) 2 weeks after the final immunization. The severity of myocarditis was determined 7 days post-infection by monitoring weight loss, conducting histopathological analysis of cardiac tissue, and evaluating cardiac enzyme markers. The control group exhibited significant weight loss, while the GEM-PA-VP1 group showed a more moderate reduction. Notably, mice immunized with GEM-Fc-VP1 demonstrated minimal weight loss, comparable to the uninfected controls (Figure 7a), indicating its strong efficacy in preventing VM.

Cardiac enzyme assays revealed that GEM-Fc-VP1 significantly reduced myocardial inflammation and damage, as evidenced by lower levels of CK and CK-MB (Figures 7b,c). Histopathological analysis corroborated these findings, showing that the PBS, GEM, and VP1 groups exhibited extensive cardiomyocyte destruction and severe lymphocytic infiltration. The GEM-PA-VP1 group displayed less severe damage, but the GEM-Fc-VP1 group showed minimal necrosis with only mild lymphocytic infiltration (Figure 7d). In parallel, the survival rates were tracked in a lethal-dose challenge experiment, where mice were monitored daily for 14 days post-infection. All mice in the PBS and GEM-PA groups succumbed by day 8, whereas the GEM-PA-VP1 group had a 40% survival rate. In contrast, the GEM-Fc-VP1 group demonstrated a significantly improved survival rate of 70% (Figure 7e). Compared to the PBS GEM, and VP1 group, the GEM-PA-VP1 and GEM-Fc-VP1 group exhibited significantly reduced expression of TNF-α, IL-1β and IL-6 in myocardial tissues, with GEM-Fc-VP1 eliciting a stronger response (Figure 7f-h). These findings highlight the superior immunoprotective potential of GEM-Fc-VP1, which effectively reduced myocardial damage and enhanced survival outcomes.

In conclusion, the GEM-Fc-VP1 vaccine outperformed GEM-PA-VP1 in all measures, significantly reducing weight loss, myocardial injury, and cardiac enzyme activity while improving survival rates. These results suggest that GEM-Fc-VP1 may offer a more effective strategy for preventing VM by limiting viral replication and inflammation within the cardiac tissue.

## Discussion

VM, primarily caused by CVB3, poses a serious threat to public health, particularly because of the lack of effective vaccines [26,27]. Despite considerable research efforts, the development of vaccines against CVB3 remains challenging. Live-attenuated vaccines, while being capable of eliciting immune responses, pose the risk of reversion to virulence, limiting their clinical application [28]. Inactivated vaccines are safer but suffer from low immunogenicity and necessitate the use of adjuvants to enhance their efficacy [29]. Furthermore, recombinant subunits [30], DNA [24], and viral vector vaccines [31], although promising, are still in the early stages of development and have not yet achieved an optimal balance between protective efficacy and safety. Notably, despite the potential of combining vector and subunit vaccines, progress has been slow. Therefore, developing a recombinant subunit vaccine that can elicit a robust mucosal immune response may represent a novel strategy for preventing CVB3 infections.

As reported in previous studies, cathelicidin antimicrobial peptides play a significant role in defending against CVB3 infection. These findings further highlight the importance of mucosal immune responses in combating CVB3 infections [32]. GEM particles offer several advantages as vaccine carriers. They activate the Toll-like receptor 2 (TLR2) pathway, promoting dendritic cell maturation and cytokine secretion, thereby enhancing antigen presentation and immune responses [33]. GEM particles can display antigens on their surfaces, facilitating antigen capture, which subsequently induces antigen-specific responses from Th cells, cytotoxic T lymphocytes, and IgA-secreting B cells, thereby initiating both local mucosal and systemic immune responses [13]. In this study, the construction and validation of PA-VP1 and Fc-VP1 recombinant plasmids demonstrated differences in expression efficiency, purification yields, and functionality. Fc fusion proteins exhibited superior solubility and stability, which are critical for maintaining antigen structure and function. The successful validation of GEM particles as antigen delivery carriers underscores their potential to induce mucosal immune responses. We successfully prepared two GEM particle vaccines: GEM-PA-VP1, based on PA-mediated antigen surface display, and GEM-Fc-VP1, based on affinity peptide-mediated antigen surface display. We then preliminarily evaluated their immunogenic effects in a CVB3-induced VM mouse model.

The GEM-Fc-VP1 group exhibited higher levels of IgG, IgG1, and IgG2a antibody levels, as well as higher neutralizing antibody titres compared to the GEM-PA-VP1 group. This finding highlights the significant advantages of Fc-fusion proteins in eliciting robust immune responses. The cytokine analysis revealed that the GEM-Fc-VP1 group was more effective in inducing both Th1 (IFN-γ, IL-2, and TNF-α) and Th2 (IL-4, IL-6, and IL-10) immune responses, suggesting a comprehensive immune activation. Assessment of myocardial enzyme activity and survival rates further indicated a marked protective effect in the GEM-Fc-VP1 group, supporting its efficacy in preventing CVB3 infections. Pathological evaluation confirmed that the role of the GEM-Fc-VP1 vaccine significantly reduced myocardial injury and inflammation. These findings suggest that Fc fusion proteins facilitate more effective traversal of mucosal epithelial cells via FcRn-mediated transcytosis, thereby promoting stronger local mucosal and systemic immune responses.

Despite these promising findings, this study has some limitations. The differences between the mouse model and human immune responses may impact the clinical efficacy of the vaccine. Moreover, the immunization dose and regimen used in this study may require adjustments for practical applications. Future studies must validate these findings in larger animal models. Additionally, clinical trials are necessary to ensure vaccine safety and efficacy.

In conclusion, GEM-Fc-VP1, based on the affinity peptide FcSP design, represents a novel mucosal vaccine delivery vector against CVB3 infection-induced VM, exhibiting notable immunogenicity and protective efficacy. By activating TLR2 signalling, GEM particles promote dendritic cell maturation and cytokine secretion [34], thereby enhancing both the humoral and cellular immune responses. Fc fusion proteins improve antigen stability and persistence through FcRn-mediated transcytosis, thereby augmenting the immune response [35]. These findings provide novel insights and a foundation for the development of effective strategies for the prevention of VM, with significant potential for clinical application. With further optimization and validation, the GEM-Fc-VP1 vaccine may serve as an effective tool for the prevention and treatment of CVB3 infections, contributing to the advancement of public health.

## Supplementary Material

CHECKLIST.pdf

## Data Availability

These sequences were derived from the public database: NCBI nucleotide. The data is publicly available on the findings of this study and unrestricted re-use is permitted via an open licence.The Data generated during the study is available at Mendeley Data at https://doi.org/10.17632/xm39sy2js7.3.
